# Ceftazidime-avibactam for the treatment of multidrug-resistant or extensively drug-resistant bacteria infection in preterm infants: case series study and literature review

**DOI:** 10.3389/fped.2025.1723315

**Published:** 2025-12-15

**Authors:** Shiqi Guang, Tian Sang, Xifang Ru, Xin Zhang, Qi Feng, Ying Wang, Shan Li

**Affiliations:** Children’s Medical Center, Peking University First Hospital, Beijing, China

**Keywords:** aztreonam, ceftazidime-avibactam, drug-resistant bacteria infection, NGS, preterm infants

## Abstract

**Objective:**

To evaluate efficacy and safety of ceftazidime-avibactam (CZA) in preterm infants with multidrug-resistant (MDR) or extensive drug-resistant (XDR) bacterial infections.

**Method:**

Retrospective analysis of clinical data was conducted on preterm infants who were monitored in NICU of Peking University of First Hospital for MDR/XDR infections between 2022 and 2025. A literature search was performed using PUBMED and WANFANG databases.

**Results:**

Six preterm infants (median gestational age 26^+3^ weeks) received CZA treatment for MDR/XDR bacterial infections, including five bloodstream infections and one ventilator associated pneumonia. CZA was used as monotherapy in three patients and as combination therapy with colistin or aztreonam in three patients. The median postnatal age at treatment initiation was 31 days. Five patients achieved clinical cure without adverse effects (AEs). One patient with concurrent CNS infection did not complete the treatment due to onset of unexpected status epilepticus. The literature search retrieved 44 preterm infants or neonates using CZA for sepsis or focal infections. Clinical cure rate was 84.09% (37/44). The most frequent AEs included liver function abnormalities, increased serum creatinine, hypomagnesemia and thrombocytopenia which were reversible.

**Conclusion:**

CZA alone or combined with aztreonam or colistin is effective and well-tolerated in critically ill preterm infants. However, the small sample size of patients limits the definitive conclusions about the safety profile of CZA, especially CNS risks. Further studies on the pharmacokinetics/pharmacodynamics and safety of CZA in preterm infants are warranted.

## Introduction

Infections caused by multidrug-resistant (MDR) or extensive drug-resistant (XDR) bacteria present a critical challenge in neonatal intensive care units (NICUs), particular for preterm infants, due to severely limited antibiotic treatment options and high associated mortality rates (1). Ceftazidime-avibactam (CZA), a novel antibiotic combination of a third-generation cephalosporin and an inhibitor for class A, C and class D β-lactamases, has been approved for complicated intra-abdominal infections, urinary tract infections and hospital acquired pneumonia caused by Gram-negative bacteria (2). While its administration in pediatric patients (3 months to 18 years old) has been approved due to similar pharmacokinetics and safety of CZA compared to those in adults (3), safety and efficacy data regarding the application of CZA in preterm infants are still under active investigation.

Notably, a recent study demonstrated CZA was well tolerated in young infants, including premature neonates with a gestational age as low as 31 weeks (4). However, this study focused on patients who received standard-of-care antibiotic therapy without confirmed MDR or XDR bacterial infections, leaving unanswered questions about the treatment efficacy of CZA and its optimal dose in preterm infants with MDR or XDR bacterial infections. Our study aims to provide more clinical evidence regarding CZA use in preterm infants with MDR or XDR bacterial infections.

## Materials and methods

This study was a retrospective case series study conducted at the neonatal intensive care unit (NICU) in Children's Medical Center of Peking University First Hospital, a tertiary-level care facility. We included 6 preterm infants who received CZA during their NICU admission between 2022 and 2025.

### CZA indications

CZA was prescribed under the following clinical scenarios:

1. Isolation of MDR/XDR pathogens from blood, tracheal aspirate, CSF, urine or peritoneal fluid cultures, supported by antimicrobial susceptibility testing.
2. Identification of pathogens with drug-resistant genes via target next-generation genomic sequencing (tNGS) of blood, tracheal aspirate, CSF, urine or peritoneal fluid samples.
3. Lack of clinical improvement despite of prior antibiotic therapy, particular carbapenems.

### Microbiology

Identification and antimicrobial susceptibility testing procedures were conducted as follows: Strains of pathogens were isolated from various specimen cultures using standard microbiology laboratory protocols. Identification of pathogens was performed using the VITEK 2 Compact System (bioMérieux, Marcy-l’ Étoile France). Antimicrobial susceptibility testing was carried out using VITEK 2 AST-N325 card (bioMérieux, Marcy-l’ Étoile France). The antibiotics tested with AST-N325 card include piperacillin tazobactam, cefoperazone sulbactam, ceftazidime, cefepime, aztreonam, imipenem, meropenem, tobramycin, amikacin, ciprofloxacin, levofloxacin, trimethoprim sulfamethoxazole, ticarcillin Clavulanic acid, doxycycline, tigecycline, polymyxin and minocycline. The Kirby-Bauer disk diffusion method was employed to assess susceptibility of CZA. A zone diameter of inhibition greater than 21 mm was interpreted as susceptible.

The operational workflow of target next-generation sequencing (tNGS) was as follows:

1. Sample processing: blood samples were centrifuged to obtain plasma; other samples including tracheal aspirate, peritoneal fluid and CSF were homogenized and centrifuged to remove debris.
2. Nucleic acids extraction and library preparation: nucleic acids were extracted from the processed samples via magnetic bead-based kits. Pathogen nucleic acids were amplified using multiplex PCR. Library construction was carried out with the automated NGS library preparation system (MatriDx Biotech Corp., Hangzhou). Library quality was assessed using the BioAnalyzer 2,100 (Agilent Technologies), and adapter ligation efficiency was verified by quantitative PCR to ensure library quality. Libraries were normalized and sequenced on the Illumina platform using a single-end 50 bp (SE50) strategy.
3. Data analysis: raw reads were filtered to remove low-quality and human sequences. Clean reads were aligned to the customed microbial database and classified using Kraken 2 (v2.1.2, confidence = 0.5). The classified microbial reads were further validated with Bowtie 2.

### Safety monitoring

During CZA treatment, potential adverse effects were monitored through serial assessments including:

1. Complete blood count
2. Liver and renal function tests
3. Blood electrolyte levels

### Data collection

Medical records were extracted by following variables:

1. Demographics: Gestational age, birth weight, sex
2. Clinical management: Apgar scores, use of mechanical ventilation, infection sites
3. Microbiological data: Culture results, antimicrobial susceptibility tests, or tNGS results
4. Treatment details: Postnatal age at CZA initiation, prior and concomitant antibiotics, CZA dosage and duration
5. Outcomes: clinical and/or microbiological response, adverse effects

### Statistical analysis

Data were analyzed using IBM SPSS Statistics software version 26. Mean, median, standard deviation, and range were calculated for descriptive data such as demographic and clinical characteristics. Frequencies and percentages were calculated for categorical variables.

### Literature review

PubMed and WANFANG databases were searched for articles with the keywords: ceftazidime-avibactam, preterm infants, and neonates. Articles published in the English and Chinese language involved pediatric patients using CZA treatment were included. Case reports and case series were individually reviewed. Clinical and microbial characteristics, CZA treatment regimens and outcomes were extracted.

## Results

Our study enrolled 6 preterm infants who received CZA treatment between 2022 and 2025 (Table 1). Five of these patients were male infants. The median gestational age was 26^+3^ weeks (ranging from 25^+6^ to 36 weeks). The median birth weight was 970 g (ranging from 800 to 2,370 g). The median Apgar score at 1 min was 6 (ranging from 1 to 10). All patients had been put on mechanical ventilation due to neonatal respiratory distress syndrome or pneumonia for various durations (ranging from 3.5 to 80 days). All patients had experienced 2 to 6 antibiotic treatments before CZA, such as meropenem (83.3%), cefoperazone-sulbactam (83.3%) and ceftazidime (33.3%). The median postnatal days when CZA started was 33 days (ranging from 6 to 117 days). The CZA dosage used in our study was 50 mg/kg (40 mg/kg of ceftazidime and 10 mg/kg of avibactam) or 62.5 mg/kg (50 mg/kg of ceftazidime and 12.5 mg/kg of avibactam) every 8 h. Three patients in our series used CZA in combination with other antibiotics to treat Gram-negative MDR/XDR infection.

**Table 1:** Demographics and clinical characteristics of included patients.

Patients	Case 1	Case 2	Case 3	Case 4	Case 5	Case 6
Gestational week	29^+1^	26^+2^	36	26	26^+5^	25^+6^
Birth weight (g)	1,300	880	2,370	980	960	800
Sex	Male	Male	Male	Male	Male	Female
Apgar scores at 1 min	6	1	10	4	8	6
Apgar scores at 5 min	9	4	10	8	10	8
Mechanical ventilation duration (days)	80	39	9	32	31	3.5
Age at CAZ (days after birth)	67	29	6	33	117	26
Infection sources	Bloodstream, IAI, VAP	VAP	Bloodstream	Bloodstream	Bloodstream, CNS	Bloodstream, pneumonia
Antibiotics before CZA	MEM, VAN, CSL, LNZ, CAZ, MTZ	MEM, VAN, AZM, CSL	CAZ, CSL, MEM, PEN	CSL, MEM, PEN, LNZ, VAN	MEM, VAN	CSL, PEN, AMC
Antibiotics concomitantly used with CZA	Colistin B	Teicoplanin	/	Aztreonam	/	Aztreonam
CZA dosage	50 mg/kg q8h	50 mg/kg q8h	50 mg/kg q8h	50 mg/kg q8h → 62.5 mg/kg q8h	50 mg/kg q8h	62.5 mg/kg q8h
CZA duration (days)	24	12	10	36	2	14
Clinical-laboratory-imaging improvement^a^	CRP decreased after 2 days	Lung infiltrate resolved after 2 days	Pneumorrhagia and septic shock resolved after 2 days, extubation achieved after 4 days	CRP decreased after 3 days, but relapsed twice upon cessation of therapy	/	CRP decreased after 3 days
Microbial eradication^b^	Blood culture turned negative after 5 days	Sputum culture remained positive, CRPA colonization	Blood culture remained negative; blood tNGS turned negative after 4 days	Blood culture remained negative; blood tNGS turned negative after 5 days and kept negative during relapses	/	Blood culture remained negative; blood tNGS turned negative after 5 days
Adverse effects	None	None	None	None	Suspicious seizures exacerbation	None
Outcomes	Cure	Cure	Cure	Cure by dose escalation to 62.5 mg/kg q8h	Treatment discontinuation	Cure

CZA, ceftazidime-avibactam; MEM, Meropenem; VAN, vancomycin; CAZ, ceftazidime; CSL, Cefoperazone Sulbactam; PEN, penicillin; LNZ, linezolid; AZM, azithromycin; MTR, metronidazole; AMC, amoxicillin-clavulanic acid.

VAP, ventilator associated pneumonia; IAI, intra-abdominal infection; CNS, central nervous system; CRKP, carbapenem-resistant Klebsiella pneumonia; ESBL-KP, extended spectrum β-lactamase producing Klebsiella pneumonia; CRPA, carbapenem-resistant Pseudomonas aeruginosa; CRECO, carbapenem-resistant Escherichia coli.

a Indicated by improved infection markers (WBC count, CRP, PCT), x-ray or vital signs.

b Indicated by negative cultures or tNGS.

Case 1 patient went through early-onset sepsis, bacterial meningitis and necrotic enterocolitis (NEC) successively. After ileostomy surgery, his CRP level raised to 180 mg/L and procalcitonin was up to 20 ng/mL. He also suffered from intractable thrombocytopenia (minimum PLT count 8 × 10^9^/L). His blood, peritoneal fluid and tracheal aspirate cultures isolated CRKP simultaneously. Considering the CRKP infection involving multiple sites and the complex history of antibiotic treatments (6 antibiotics used in early postnatal days), CZA was used in combination with colistin. A loading dose of colistin at 2 mg/kg was given, followed by a maintenance dose of 1.5 mg/kg every 12 h. He had clinical improvement in 3 days and microbial eradication in 5 days of treatment. We monitored his liver and renal functions within his baseline levels; his PLT count gradually recovered.

Case 2 patients experienced prolapse of cord during his birth and thus suffered from severe neonatal asphyxia. He could not be weaned from mechanical ventilation due to frequent convulsion attacks. During the treatment process, the patient showed worsening respiratory status that required increased ventilatory support. The chest radiography showed newly emerging pulmonary infiltrates. His condition did not improve after empirical treatment of Cefperazone-Sulbactam. Since tracheal aspirate culture reported heavy growth of CRPA, we started CZA (50 mg/kg IV q8h) treatment. His condition significantly improved and extubation was achieved in 10 days. His PLT level, as well as liver and renal function were normal during the whole treatment course which lasted for 12 days.

Three of our patients were successfully diagnosed by target next-generation sequencing (tNGS) when their blood cultures stayed negative (Case 3, 4 and 6). Case 3 patients manifested ARDS after birth, and his conditions rapidly worsened to septic shock and pneumorrhagia under treatment of meropenem. Blood tNGS detected CRECO with *blaGES* gene (Table 2), leading our antibiotic choice to CZA. His vital signs stabilized soon, and extubation achieved within 4 days of CZA treatment. tNGS detected carbapenem resistant pathogens carrying blaNDM-1 gene in Case 4 and Case 6 patients (Table 2). Thus, we used the combination of CZA and aztreonam (ATM) for both patients. CZA and ATM (30 mg/kg) were infused simultaneously using 3-way stopcock every 8 h for 3 h. Case 4 patient experienced 2 episodes of recurrence of elevated infection markers upon cessation of therapy when he used 50 mg/kg of CZA every 8 h, but his infection was adequately controlled after dose escalation to 62.5 mg/kg every 8 h. Based on our clinical experience, we applied 62.5 mg/kg of CZA and 30 mg/kg of aztreonam every 8 h for Case 6 patient who showed a rapid clinical response and was able to cease the medication uneventfully.

**Table 2:** Microbiological characteristics of included patients.

Patient	Sample	Detection method	Pathogen	Antimicrobial susceptibility/drug resistant gene
Case 1	Blood, peritoneal fluid. tracheal aspirate	Culture	CRKP	R to TZP, CSL, CAZ, FEP, ATM, IPM, MEM, CIP, LVX
				I to MNO
				S to **CZA**, TOB, AMK, SXT, DOX, TGC
Case 2	Tracheal aspirate	Culture	CRPA	R to IMP, MEM, CAZ, CSL, TCC
				I to FEP
				S to **CZA**, LVX, FEP, AMK, TOB
Case 3	Blood	Culture negative, tNGS positive	CRECO	blaGES gene
Case 4	Blood	Culture negative, tNGS positive	CRKP	*Ctx* and *Ndm* gene
Case 5	Blood, cerebral spinal fluid	Culture	ESBL-KP	R to TZP, CSL, CAZ, ATM, MNO, DOX
				I to LVX
				S to **CZA**, FEP, MEM, IPM, TOB, AMK, CIP, SXT, TGC
Case 6	Blood, tracheal aspirate	Blood tNGS, tracheal aspirate culture	CRECO	R to TZP, **CZA**, CSL, TCC, CAZ, FEP, ATM, IMP, MEM, TOB, LVX, CIP, SXT, DOX, MNO
				I to AMK, POL
				R to TGC

R, resistant; I, intermittent; S, sensitive.

TZP, piperacillin tazobactam; CSL, Cefoperazone Sulbactam; CAZ, Ceftazidime; FEP, Cefepime; ATM, Aztreonam; IPM, Imipenem; MEM, Meropenem; TOB, Tobramycin; AMK Amikacin; CIP, Ciprofloxacin; LVX, Levofloxacin; SXT, Trimethoprim Sulfamethoxazole; TCC, Ticarcillin Clavulanic acid; DOX, Doxycycline; TGC, Tigecycline; POL, polymyxin; MNO, minocycline.

We also reported one patient (Case 5) who was not able to complete the CZA treatment. This patient was diagnosed with CNS fungal infection with severe hydrocephalus and progressively expanding cerebral cyst that required to place Ommaya reservoirs to drain. After the drainage surgery, he had ventriculitis and meningitis caused by ESBL-KP that did not respond well to meropenem. We attempted to use CZA for the patient but unexpected status epilepticus occurred after 2 days’ treatment despite his CSF indicators getting better. We withdrew the CAZ treatment and his seizures subsided with no recurrence.

## Discussion

In low- and middle-income countries, Gram-negative bacteria account for one-half to two-thirds of neonatal sepsis cases, with particularly high rates of multidrug resistance observed in *Acinetobacter* spp, *Klebsiella* spp and *E coli* (5, 6). The risk factors of MDR/XDR infections in NICUs include lower birth weight, premature rupture of the membrane lasting for more than 18 h, lower Apgar scores, longer hospital stays, as well as the use of ventilator (7), which means preterm infants are highly vulnerable to the infections caused by carbapenem-resistant Gram-negative bacteria (CRGN). CRGN infections have posed a substantial threat to the neonatal healthcare system due to limited development of new antibiotic agents and high rates of morbidity and mortality in NICU patients (8, 9). CZA is a novel combination of cephalosporin and β-lactamase inhibitor. It is now considered to be safe and effective to treat complicated and severe infections in pediatric patients aged from 3 months and 18 years (3). Current available data regarding the use of CZA in premature infants is still limited.

Our literature search retrieved 10 articles including 44 patients using a total of 54 courses of CZA during their stay in NICU (Table 3). Of these patients, 42 (95.45%) were premature (15 extremely premature, 16 very premature and 11 moderate to late premature) infants. Thirty-seven patients (84.09%) were eventually cured, but 7 patients (15.91%) died on account of severe infection or related complications. One patient who used CZA three times developed drug resistance. Among all treatment courses, more than half (31/54, 57.41%) were used due to bloodstream infections including 21 CRKP, 7 ESBL-KP infections and 3 infections of carbapenem-sensitive pathogens. Twelve courses (12/54, 22.22%) were used with diagnosis of culture-negative sepsis. Eight courses (14.81%) were commenced to treat focal infections including 3 urinary tract infections, 3 NEC or NEC-like cases, 1 ventilator-associated pneumonia and 1 osteoarthritis. Besides, there were 3 short-term courses (4–5 days) applied for previous CRKP infection or CRKP colonization. Thirty-nine of 54 (72.22%) treatment courses lasted for more than a week with maximum treatment duration being 58 days. The treatment dosage of reported cases varied substantially from 25 to 95 mg/kg every 8 h (for CZA treatment). Within this range, the most frequently used dosages included 62.5 mg/kg (21/54, 38.89%) and 50 mg/kg (17/54, 31.48%) every 8 h. The rate of CZA adverse effects (AEs) in these reported premature infants was up to 31.48% (17 of 54 courses). The most frequent AEs included liver function abnormalities (elevated ALT, AST, γ-GGT or directed bilirubin), increased serum creatinine, hypomagnesemia and thrombocytopenia. All these AEs were reversible and do not require treatment discontinuation.

**Table 3:** Summarization of clinical data of currently reported premature infants and neonates that used CZA treatment.

References	Patient ID	SEX	GW	Birth weight/g	Index infection	Microorganism	Age at CAZ (days after birth)	Duration of CZA	Dosage of CZA (mg/kg q8h)	AEs	Outcome
2019, Esposito P (10)	1	NR	NR	860	BSI, meningitis	CRKP	39	47	95 (75/20) → 31.5 (25/6.5) due to thrombocytopenia	thrombocytopenia	Cured
2019, Iosifidis (11)	2	F	29^+6^	920	BSI	CRKP	23	14	65 (52/13)	Neg	Cured
	3	M	29^+6^	1,260	BSI	CRKP	15	9	62.5 (50/12.5)	DBIL increase, hypomagnesemia	Cured
	4	F	30^+3^	1,540	/	previous CRKP BSI and CRKP colonization	55	5	32 (28/7)	Neg	Cured
	5	M	32^+4^	2,030	BSI	CRKP	135	21	62.5 (50/12.5)	hypomagnesemia	Cured
	6	M	25^+5^	900	/	CRKP colonization and susceptible S. marcescens	51	4	62.5 (50/12.5)	Neg	Cured
	7	M	37^+3^	2,440	BSI	CRKP	25	14	62.5 (50/12.5)	Neg	Cured
2020, Coskun (12)	8	M	27	1,000	UTI	CRKP	25	10	50 (40/10)	glycosuria	Cured
2022, Nascimentoc (13)	9	M	29	830	BSI	CRKP	46	14	50 (40/10) → 23.75 (19/4.75) q48h due to peritoneal dialysis → 50 (40/10)	NR	Cured
2022, Asfour (14)	10	F	27	920	BSI, meningitis	CRKP	17	21	62.5 (50/12.5)	NR	Cured
	11	F	28	925	BSI	CRKP	56	5	62.5 (50/12.5)	Cr increase	Death
2023, Mangarov (15)	12	M	36	3,220	BSI	CRKP	28	17	50 (40/10)	NR	Cured
2023, Marino (16) 2023, Pu (17)	13	M	25	590	BSI	ESBL-KP	35	14	50 (40/10)	Neg	Cured
	14	M	25^+3^	940	BSI	CRKP	19	15	50 (40/10)	Neg	Cured
	15	F	25^+3^	590	BSI	ESBL-KP	7	7	50 (40/10)	Neg	Cured
	16	F	28^+4^	1,230	BSI	ESBL-KP	16	14	50 (40/10)	Neg	Cured
	17	F	29^+4^	1,260	BSI	ESBL-KP	22	15	50 (40/10)	Neg	Cured
	18	F	24^+^5	650	BSI	ESBL-KP	8	11	50 (40/10)	Neg	Cured
	19	M	29^+5^	1,450	BSI	ESBL-KP	4	18	50 (40/10)	Neg	Cured
	20	F	30^+4^	940	BSI	ESBL-KP	13	11	50 (40/10)	Neg	Cured
	21	F	34^+4^	2,400	BSI, osteoarthiritis	CRKP	50	14	50 (40/10)	Neg	Cured
	22	M	32^+4^	1,270	Osteoarthritis	CRKP	49	28	50 (40/10)	Neg	Cured
2024, Ftergioti (18)	23	M	34^+4^	2,380	BSI	CRKP	10	30	65 (52/13)	GGT increase	Cured
					/	Previous CRKP BSI	65	5	62.5 (50/12.5)	GGT increase	Cured
	24	M	26^+6^	430	NEC, peritonitis	CRPA	29	15	42.5 (34/8.5)	AST ALT GGT increase	Cured
					sepsis	culture negative	44	7	53.8 (43/10.8)	Neg	Death
	25	M	25^+6^	760	BSI	CRKP,CRRA	19	4	37.5 (30/7.5)	Neg	Death
	26	M	33^+5^	1,490	sepsis	culture negative	80	4	50 (40/10)	Cr increase	Cured
					sepsis	culture negative	112	10	62.5 (50/12.5)	Neg	Death
	27	M	30^+2^	1,180	BSI	CRKP	7	58	62.5 (50/12.5)	AST ALT DBIL increase, thromnocytopenia	Death
	28	F	29^+5^	1,315	sepsis	culture negative	12	3	62.5 (50/12.5)	AST increase	Cured
	29	M	29^+2^	880	BSI	CRKP	200	3	62.5 (50/12.5)	AST ALT increase	Cured
	30	M	28^+2^	1,170	NEC	culture negative	46	26	62.5 (50/12.5)	Neg	Cured
	31	M	NR	NR	sepsis	culture negative	31	12	50 (40/10)	hypomagnesemia, hypocalcemia, ALT AST GGT increase	Cured
	32	F	31^+6^	1,680	sepsis	culture negative	15	4	25 (20/5)	Neg	Cured
					sepsis	culture negative	114	6	62.5 (50/12.5)	thrombocytopenia	Cured
	33	M	25^+5^	850	sepsis	culture negative	14	35	62.5 (50/12.5)	hypomagnesemia, AST ALT DBIL increase	Cured
					BSI	CRKP	109	9	62.5 (50/12.5)	Neg	Drug resisance
					sepsis	culture negative	175	10	50 (40/10)	Neg	Cured
	34	M	30^+5^	1,675	BSI	CS SM, E.coli	120	3.5	62.5 (50/12.5)	Neg	Cured
	35	M	26^+1^	510	BSI	CSKP	31	10	25 (20/5)	AST ALT DBIL increase, hypomagnesemia	Cured
	36	F	27^+5^	580	sepsis	culture negative	23	6	56.3 (45/11.3)	Neg	Death
	37	F	28^+3^	730	BSI	CSKP	96	5	NR	Neg	Cured
					BSI	CRKP	183	14	47.5 (38/9.5)	Neg	Cured
					sepsis	culture negative	225	9	62.5 (50/12.5)	Neg	Cured
					VAP	CIPA	277	20	NR	Neg	Cured
	38	M	31^+1^	2,440	BSI	CRKP	26	16	62.5 (50/12.5)	Neg	Cured
	39	M	27^+2^	940	sepsis	culture negative	11	7	67.5 (54/13.5)	Neg	Death
	40	M	24	650	BSI	CRKP	12	3	25 (20/5)	Neg	Cured
	41	M	34^+6^	2,620	BSI	CRKP	149	18	62.5 (50/12.5)	Neg	Cured
	42	M	32^+3^	1,180	NEC-like	culture negative	34	8	61.3 (40/12.3)	AST ALT DBIL increase	Cured
	43	M	34^+4^	NR	UTI	CRKP	18	24	66.3 (53/13.3)	Neg	Cured
					UTI	CRKP	57	10	62.5 (50/12.5)	Neg	Cured
2022, Nie (Chinese) (19)	44	M	34^+4^	2,280	BSI	CRKP	14	15	50 (40/10)	Neg	Cured

GW, gestational week; CZA, ceftazidime-avibactam; BSI, blood stream infection; UTI, urinary tract infection; Neg, negative; NR, not reported; VAP, ventilator associated pneumonia; CRKP, carbapenem resistant Klebsiella pneumonia; CRPA, carbapenem resistant Pseudomonas aeruginosa; ESBL-KP, extended spectrum β-lactamase producing Klebsiella pneumonia; CSSM, carbapenem sensitive serratia marcescens; DBIL, direct bilirubin; AST, aspartate aminotransferase; ALT, alanine transaminase; GGT, gamma-glutamyl transpeptidase.

A recent pharmacokinetics study supports CZA use in infants <3 months (minimum gestational age 31 weeks) with recommended doses: 37.5 mg/kg q8h for >28 days, 25 mg/kg q8h for ≤28 days (4). However, this study did not include patients with MDR/XDR infections or preterm infants younger than 31 gestational weeks, leaving critical gaps in our understanding of CZA optimal dose and effectiveness for preterm infants with MDR/XDR infections. It is worth noting that CZA is susceptible to the inoculum effect (defined as attenuated antibacterial activity with increased inoculum size), which may lead to the failure to treat patients with a heavy bacterial load (20, 21). Recent studies have demonstrated that the current dosing regimen of CZA is insufficient for severe infection patients. Increasing dosage or prolonging infusion time have been adopted to achieve better efficacy (22). Hence, adequacy of CZA dose in preterm infants with severe XDR/MDR infection remains a question to discuss.

In this paper, we reported 6 premature infants who received CZA as the monotherapy or combination treatment for Gram-negative MDR/XDR infections. Given the complex infection and medication histories of Case 1 patient, we chose CZA combined with colistin for synergetic effect. This combination has been reported to show potential efficacy in difficult clinical situation (23, 24). This patient underwent intractable thrombocytopenia (minimum PLT count 8 × 10^9^/L) during the CRKP infection. Although there were case reports mentioning that thrombocytopenia is an adverse effect of CZA, we started CZA on this patient. His PLT count gradually recovered with the infection improving, which means thrombocytopenia is not contradicted to CZA. Besides, this patient had parenteral nutrition associated cholestasis before CZA treatment. We did not observe exacerbation of his liver function after using CZA.

Generally, the identification of microorganisms causing sepsis by culture-dependent methods takes days, delaying the orientation of targeted treatment. Furthermore, the culture positivity rate remains suboptimal. Next-generation sequencing (NGS) provides better sensitivity and specificity for pathogen identification compared to traditional methods, especially in the scenario with negative cultures (25). Thus, its clinical applications are becoming increasingly widespread. In our study, blood cultures were negative in three patients with suspected sepsis (Case 3, 4 and 6), yet tNGS successfully detected pathogenic organisms and drug resistance genes. Particularly worth mentioning is the detection of blaNDM-1 drug-resistant gene in both Case 4 and Case 6 patients. Aztreonam is exempt from hydrolyzation by class B metallo-β-lactamases (MBLs) encoding by blaNDM-1 gene, but it cannot escape from ESBL or carbapenemase which are produced by most MBL-strains. Therefore, the association of CZA and ATM restores the antimicrobial activity of ATM. This combination therapy demonstrated significant synergy against XDR pathogens with the potential to improve clinical outcomes in critically ill patients (26, 27).However, Case 4 patient experienced infection relapses twice upon ceasing the treatment when he used 50 mg/kg of CZA plus 30 mg/kg of ATM every 8 h. He was completely cured by increasing the CZA dosage to 62.5 mg/kg every 8 h, which optimized the ratio of ATM and avibactam. To the best of the authors’ knowledge, reports about CZA-ATM treatment in preterm are scarce. Our study provides valuable real-word experience about CZA-ATM dosage optimization in preterm infants. Clinical efficacy of CZA-ATM for preterm infants with MDR/XDR infections warrants larger randomized controlled trails.

For the patients with negative blood cultures but positive tNGS results, we chose to repeat tNGS tests at 4–5 days after CZA treatment to monitor microbial eradication. Multiple studies have shown the half-life of foreign nucleic acids circulating in the blood including microbial DNA, fetal DNA and tumor-derived DNA is rather short as 2–3 h (28–30). A recent study also showed persistent positive NGS results indicated microbial resistance and failure of initial empirical antibiotic treatment and also predicted septic shock and adverse outcome (31). Conversely, the conversion of an NGS result from positive to negative can serve as a valuable biomarker for a positive treatment response.

Here, we also reported Case 5 patient who failed to continue CZA treatment due to unexpected status epilepticus during the treatment. Antibiotic-induced epileptic seizures have drawn growing attention in recent years. Cephalosporins can act as competitive antagonists of the gamma-aminobutyric acid (GABA) receptor or agonists of glutaminergic N-methyl-D-Aspartate (NMDA) receptor to trigger seizures (32). Ceftazidime was reported to cause generalized convulsions in a brain tumor patient when it was used preoperatively (33). A retrospective pharmacovigilance study using disproportionality analysis revealed that compared to other antibiotics (ceftazidime, ceftriaxone, meropenem and imipenem), CZA was associated with higher reporting of neurological AEs, such as encephalopathy, seizures, coma, mental impairment and myoclonus (34). The risk factors related to neurotoxicity of cephalosporins including reduced renal clearance, increased CNS penetration (CNS infections, underlying brain abnormality), extremely high dose and increased unbound form of the drum (hypoalbuminemia) (32, 34). For our Case 5 patient, we hypothesized that his severe CNS infection and structural lesions damaged the blood-brain barrier, leading to the increased drug concentration in CSF. Furthermore, he also had focal epileptiform discharges detected by vEEG, which might also predispose him to neurotoxicity of CZA. Although his status epilepticus subsided after we withdrawn CZA, a definitive causal relationship between CZA and the CNS adverse effect could not be established without measuring drug concentration in CSF and serum. Prospective therapeutic drug monitoring is warranted to better understand the pharmacokinetics and safety profile of CZA in preterm infants. In the meantime, we could enhance the monitoring of CNS conditions for patients with CNS abnormalities who used CZA.

In conclusion, for preterm infants who had infections caused by Gram-negative MDR/XDR bacteria, CZA as monotherapy or combined with ATM/colistin offer favorable clinical and microbiological responses. Currently reported AEs, such as liver and renal function damages, thrombocytopenia, are reversible after treatment ends. The primary limitation of this study is the small sample size from a single center, which restricts the generalizability of our findings. To provide more definitive evidence on efficacy, optimal dosing, and safety, future research must involve larger, multi-center cohorts.

## Data Availability

The original contributions presented in the study are included in the article/Supplementary Material, further inquiries can be directed to the corresponding author
